# Xia-Gibbs Syndrome: A Review of Literature

**DOI:** 10.7759/cureus.12352

**Published:** 2020-12-29

**Authors:** Chanan Goyal, Waqar M Naqvi, Arti Sahu, Ashish S Aujla

**Affiliations:** 1 Physiotherapy, Datta Meghe Institute of Medical Sciences, Wardha, IND; 2 Paediatric Neurology, Kids Care Paediatric Neurology Center, Raipur, IND

**Keywords:** xia-gibbs syndrome, rehabilitation, rare genetic disorder, whole-exome sequencing, paediatric neurology, growth hormone replacement therapy, neurodevelopmental treatment, physiotherapy

## Abstract

Xia-Gibbs syndrome (XGS) is a rare genetic disorder that has been discovered as a distinct clinical entity in the recent past. The occurrence has been attributed to the mutation of AT Hook DNA binding motif Containing 1 (AHDC1) gene that is carried on chromosome 1p36. The concerned gene participates in deoxyribonucleic acid (DNA) repair apart from other crucial functions. The mutation results in dysfunction that leads to neurodevelopmental delay. The spectrum of manifestations constitutes intellectual disabilities, hypotonia, expressive language delay, sleep difficulties, and short stature. Dysmorphic facial features include depressed nasal bridge, hypertelorism, down-slanting or up-slanting palpebral fissures, horizontal eyebrows, dysplastic dentition, thin upper lip vermilion, and micrognathia. The phenotype is still expanding. The condition may range from mild to severe dysfunction depending on the area and site of genetic aberration but variation is evident. Thus, the correlation between genotype and phenotype is largely unclear. XGS should be considered as a differential diagnosis for patients presenting with intellectual as well as developmental disabilities. Whole-exome sequencing (WES) is the genetic test that is largely used for the confirmation of diagnosis. Less is known about the natural history as only a few adults with XGS have been documented in the literature. Age-appropriate cancer screening is recommended for patients with XGS as the gene mutation alters DNA repair mechanisms that may trigger tumour formation. The management of patients diagnosed with XGS is an area that needs investigation. Though use of growth hormone replacement therapy and physiotherapy intervention have been reported as effective in previous studies, research on effective means of care of these patients is warranted on a larger number of patients. We present a review of current literature on what is known about XGS that would facilitate to identify knowledge gaps for paving a way for further studies. This, in turn, will help in provision of early and effective rehabilitation services for patients with XGS.

## Introduction and background

Xia-Gibbs syndrome (XGS) is a rare genetic disorder that occurs due to a heterozygous truncating mutation of a gene on chromosome 1p36 [1,2]. XGS results from mutations in the AT-Hook DNA binding motif Containing 1 (AHDC1) gene, which encodes a transcription factor for binding of deoxyribonucleic acid (DNA). Being a neurodevelopmental disorder, it is characterized by intellectual impairment, structural anomalies of the brain, hypotonia, delayed global development, feeding issues, sleep difficulties, facial dysmorphism, and short stature [1-4]. The range of difficulties makes it imperative for effective management to be multidisciplinary.

XGS was identified as a distinct clinical entity in 2014 by Xia and colleagues. On performing genetic tests by whole-exome sequencing (WES) in four patients with clinical features of intellectual impairment, low tone, dysmorphic facies, delayed language development, and sleep issues, they found a new, dominant, monoallelic mutation of the AHDC1 gene [1]. In 2015, WES testing was performed on more than 2000 patients who presented with developmental delay (DD) and/or intellectual disability (ID) by Yang et al. They identified mutations same as those found by Xia et al. in seven patients [2]. These seven patients were diagnosed with XGS due to similar clinical findings. They attributed haploinsufficiency of 1p36.11 situated in the AHDC1 gene as the cause of XGS because it encodes a protein made of 1603 amino acids [2,5]. They remarked that the mutation results in functional loss of this protein which plays a vital role in epigenetic and transcriptional regulation in the embryo and during axonogenesis [6-8]. This links the genotype with neurodevelopmental delay in the phenotype. 

As it was discovered less than a decade ago, the information base about XGS is in the nascent stage. Though there are studies that provide the details of genotype and phenotype of this condition, the small number of cases under investigation is a major limitation in most studies. There is a paucity of literature on the rehabilitative care of patients with XGS. We present a review of currently available literature on XGS that will serve as a foundation for looking at gaps in knowledge and to recommend areas of investigation for further studies.

## Review

PubMed and ScienceDirect databases were searched with keywords Xia-Gibbs syndrome, XGS and AHDC1. Relevant studies of English language were taken into account for the review.

Prevalence

Being a recently found disorder, the prevalence of XGS is difficult to determine [9]. Approximately 100 cases have been reported around the globe since its discovery in 2014 [10]. According to a study published in January 2020, less than 50 patients have been described in the literature since its discovery in 2014 [3]. The incidence of XGS in the general population was estimated to be less than one in 1,000,000 live births [11]. WES has been used to identify the new cases because the clinical manifestations of XGS are variable, and the phenotype is still expanding [9,12].

Diagnosis

Out of thousands of genetic disorders, it is challenging to make a clinical diagnosis for most conditions except the common ones like Down syndrome that present with classical facial features. The rare diseases when suspected may need a range of genetic tests for confirmation [13]. Moreover, in developing countries like India, more often than not, it is not feasible to get genetic testing done due to financial constraints. Frequently, children do not receive an accurate diagnosis which hinders understanding of prognosis. Subsequently, lack of early intervention leads to decrease in probability of achieving maximum potential through treatment and rehabilitation [14].

In comparison to karyotyping, while array comparative genomic hybridization (aCGH) provides better rate of diagnosis of about 20%, WES has shown even more sensitivity to reveal the gene mutation in 60% of persons with ID and/or DD of unexplained nature [15,16]. When the history and clinical evaluation of an individual point towards a genetic cause, but the phenotypic characteristics do not match with the involvement of a particular gene that can cause the disorder or when high genetic variability is demonstrated by the phenotype for which specific test is unavailable or when the available phenotype-specific genetic test is inconclusive, WES test is recommended by American College of Medical Genetics [17,18]. Moreover, in the study conducted by Yang et al., all cases of XGS were identified by WES [2]. In the recent past, WES has enhanced detection of genes implicated in diseases of unclear etiology and in understanding the heterogeneous nature of genes [19].

Owing to its establishment as a specific condition, XGS must be included as a differential diagnosis for persons with ID and/or DD who present with associated facial dysmorphism, hypotonia, and apnea while asleep.

Genotype

XGS (Online Mendelian Inheritance in Man #615829) is an autosomal dominant expression of AHDC1 mutation [4]. AHDC1 includes hooks that are obligatory in binding of DNA [20]. AHDC1 is expressed in early embryonic life in the mouse brain, revealing that it has a pivotal role in the development of the brain [5]. Features can mimic Muenke syndrome which is also autosomal dominant [21]. The de novo variant of AHDC1, which has been associated with XGS, is found to be close to the terminal region ‘N’ [22]. Out of seven exons, exon six encodes over 1600 amino acids and contains a couple of conserved regions that participate in structural modification of DNA, signal transmission of ions, transfer of fluids and electrolytes [4,20,23,24].

AHDC1 includes domains that when mutated can cause ID and diminish DNA repair [25]. The gene variant associated with XGS has also been found to be similar to chronic kidney disease (CKD)-associated gene variants [26]. Repair of damaged DNA and interaction with proteins in neural development have been attributed as functions of AHDC1 in earlier studies [8,20,27-29]. Inadequate amount and function of the developmental protein encoded by AHDC1 adversely affect the developing brain [5]. The functional importance of the AHDC1 gene indicates that the copy number gain can lead to the phenotypic characterization of patients with XGS [4]. This is interesting because a genotype-phenotype link between AHDC1 mutation and occurrence of XGS is indicated. Cardoso-dos-Santos (2020) suggested that the investigation on interactions of AHDC1 that are implicated in neurocognitive development in humans can lay a firm foundation for the conclusion of the mechanisms underlying XGS [22]. Also, it can be derived that variants close to the C-terminal region result in a severe form of disorder and the ones closer to the N-terminal region express a milder form [12]. As an exception to this, Ritter et al. (2018) reported a case of an individual with ID that was not severe as evidenced by his ability to speak three languages had a mutation close to C-terminus [30]. Another exception is a case documented by Murdock et al. (2019) who presented with a severe form of ID yet was found to have a mutation situated at the beginning of AHDC1 [31]. It is noteworthy that genetic defects that disrupt DNA repair pose an increased risk of cancer. Though the exact role of AHDC1 in humans is still a matter of debate, it is recommended that patients diagnosed with XGS should be referred to specialists for cancer screening while keeping the suspicion high [31].

Phenotype

Damage to DNA, which contains the blueprint of protein synthesis, influences and ultimately alters brain development, consequently painting the clinical picture of XGS [5,32]. The striking features of XGS are cognitive impairment, delayed development, expressive language delay, brain structure anomalies, low tone, dysmorphic facies, vision issues, and poor quality sleep [1-4,9,12,13,33-35]. Sleep apnea has been described in many patients with polysomnographic confirmation. Besides these primary manifestations, clinical presentation may include behavioural abnormalities, autistic features, and seizures [2,4,12,30]. Although aggression is not a common finding, self-injurious behaviour has been reported in a few children diagnosed with XGS [2,4,9,12]. In August 2020, Goyal et al. also reported unilateral ptosis and scoliosis in an Indian child diagnosed with XGS [35]. Dysmorphic features of the face include depressed nasal bridge, hypertelorism, downslanting or upslanting palpebral fissures, horizontal eyebrows, dysplastic dentition, thin upper lip vermilion, and micrognathia [1,4,9,12,13,30,35].

In more than half of the patients, brain abnormalities like corpus callosum hypoplasia or dysgenesis, delayed myelination or hypomyelination, leukomalacia, dysmorphic sulci-gyri, and cystic lesion, have been observed as magnetic resonance imaging (MRI) findings [1-3,12,13,30,33-35]. Movement disorders including ataxia, tremors, and bradykinesia are frequently associated with XGS [2,4,31]. It needs further investigation to include movement disorders in classical features of XGS. Gumus et al. observed brachycephaly, microcephaly, craniosynostosis, and seizures apart from the usual spectrum of presentation in their case report of the first patient from Turkey that was documented [3]. Yang et al. remarked that out of their reported seven patients with XGS, one had sagittal craniosynostosis too [2,36]. Cardoso-dos-Santos et al. reported a detailed clinical description of the first XGS case in Brazil that immensely contributed to correlate its genotype and phenotype [22].

XGS in adults

Genetic disorders are typically described in the paediatric population as they are usually identified in childhood. Out of approximately 100 cases reported since the discovery, the majority were under 10 years of age and a very few were in their third decade of life [12,13,30]. This reflects that the knowledge about the natural history of this condition is obscure. The eldest patient reported was a 55-year-old male. Unlike 90% of cases described earlier, Murdock et al. (2019) documented that this adult did not have low tone but he displayed remarkable disturbance in coordination and balance as evidenced by ataxia and frequent falls [31]. The frequent finding of sleep apnea was also missing though disruption of sleep was recorded. Surprisingly, he had prominent macrocephaly which was novel for XGS, though it is sometimes associated with a craniosynostosis condition known as Muenke syndrome [21,31]. Scoliosis was reported in one-fifth of the surveyed patients with XGS, which is a much higher prevalence than the general population [12]. Scoliosis can be attributed to connective tissue abnormalities as the 55-year-old male also carried loose and soft skin texture [31]. Additionally, another cohort study found that almost half of the cases with XGS had an abnormality in the skin and in other connective tissues [30,37]. Therefore, prompt identification and management of scoliosis are warranted to avoid further complications. Behavioural problems in adults like impulsiveness, aggression, self-mutilation, and inadequate interaction in society were first observed in some XGS patients [2,12]. On attaining adulthood, screening for medical conditions like hypertension should be carried out [31].

Therapeutic interventions

Role of Growth Hormone Replacement Therapy

Cheng et al. (2019) reported two cases from China who presented with short stature. On endocrinological work-up, they were found to be deficient in growth hormone [38]. Before the confirmatory genetic testing, growth hormone was used therapeutically. Significant improvement was recorded with a growth velocity of approximately 10 centimeters per year in both children. No side effects of the treatment were noticed during the study. This study demonstrated the beneficial effect of replacing growth hormone in patients with XGS [38]. Nonetheless, further evaluation into the matter is required to understand the long-term effects as well as the consistency of the effect of this treatment.

Role of Physiotherapeutic Intervention

Physiotherapy is well established as being crucial in treatment of motor delay due to various etiology. A study conducted by Goyal et al. (2020) demonstrated tremendous improvement in gross motor skills of a child with XGS [35]. At the time of presentation, he was two years old and was unable to sit without support. After regular physiotherapy sessions based on the principles of neurodevelopmental treatment and sensory integration, he was not only able to sit and stand without support but could also ambulate independently at about three years of age. Unstable surfaces like equilibrium boards, Swiss ball, and swings were used to improve balance through vestibular stimuli. Proprioception was provided through weight-bearing exercises, wheelbarrow and joint compressions. Mild scoliosis was dealt with gentle rib cage mobilization, facilitation of side flexor muscles of the trunk, and supported hanging. Trunk stabilizing pressure input orthosis (SPIO), bilateral ankle-foot orthosis (AFO), and a rollator walker were used during the course of rehabilitation [35]. This physiotherapy intervention significantly increased the child's participation in society. Parents of children with XGS should be prompted for regular physiotherapy for gain in function.

## Conclusions

The complete picture of the genotype-phenotype correlations are still unclear and are being investigated globally as is evident in the growing literature. We recommend delving into the part played by the AHDC1 gene in humans to establish links with neurocognitive development in persons affected with XGS. The spectrum of clinical manifestations is not all-inclusive. The variations in presentations indicate a need to differentiate the phenotype that can be deemed typical for XGS from features that can be termed as chance associations. Furthermore, this would help in suspecting a diagnosis of XGS based on clinical evaluation and other investigations before undertaking genetic testing. Consequently, early intervention can be triggered. Delineation of natural history is warranted by documentation of follow up of individuals with XGS. The area of least knowledge is the management or care of these patients. Screening for cancer and CKD is recommended due to vulnerability of the population as evident from the literature. Though growth hormone replacement therapy and physiotherapy have been reported to be beneficial in individual cases, standardized effective management for rehabilitation of these patients by a multidisciplinary team is undocumented and requires exploration.
